# Author Correction: Accurate holographic light potentials using pixel crosstalk modelling

**DOI:** 10.1038/s41598-023-44866-1

**Published:** 2023-10-17

**Authors:** Paul Schroff, Arthur La Rooij, Elmar Haller, Stefan Kuhr

**Affiliations:** Department of Physics, SUPA, University of Strathclyde, Glasgow, G4 0NG UK

Correction to: *Scientific Reports* https://doi.org/10.1038/s41598-023-30296-6, published online 24 February 2023

The Code Availability section in the original version of this Article was omitted. It now reads:

“The code used to generate the results in this work is available on GitHub, https://github.com/paul-schroff/hologradpy.”

The original Article has been corrected.

