# Supplementary material for: Accurate holographic light potentials using pixel crosstalk modelling
Source: Sci Rep. 2023 Feb 24;13:3252. doi: 10.1038/s41598-023-30296-6 (PMC9958060; doi:10.1038/s41598-023-30296-6)
Supplement: Supplementary file 1 — Supplementary Information. [file 41598_2023_30296_MOESM1_ESM.pdf]

Supplementary information

Accurate holographic light potentials using pixel crosstalk modelling

Paul Schroff[1], Arthur La Rooij[1,*], Elmar Haller[1], and Stefan Kuhr[1]

[1]Department of Physics, SUPA, University of Strathclyde, Glasgow G4 0NG, United Kingdom
[*]arthur.larooij@strath.ac.uk

Experimental setup

Light at wavelength $\lambda = 852\,\mathrm{nm}$ from a single-mode fibre is collimated by a triplet lens (Melles Griot 06 GLC 001) with a specified wavefront distortion of $< \frac{\lambda}{4}$ and is expanded by a telescope (Thorlabs GBE10-B) to a diameter of 9.4 mm at the SLM. The light is polarised along the horizontal plane by a polarising beam splitter (see Fig. S1). The beam is reflected by the SLM (Hamamatsu X13138-07, 12.5 µm pixel pitch, 1272×1024 pixels) at an angle of $\sim 10^\circ$ and is focussed onto the camera (Matrix Vision mvBlueFOX3-1012dG, 3.75 µm pixel pitch, 1280×960 pixels) by the Fourier lens (Thorlabs ACT508-250-B).

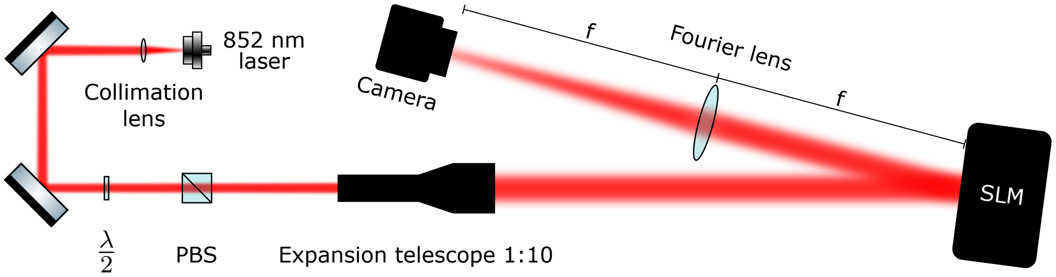

Figure S1. Schematic of the experimental setup.

Wavefront measurement

To generate experimental light potentials that match the simulated ones, it is essential to precisely know the wavefront of the light reflected by the SLM and the intensity profile of the incident laser beam. We measure the constant phase, φ_C, across the SLM using a scheme introduced in a previous study by Zupancic et al.[S1]. To measure the intensity profile across the SLM, we sample the local intensity by displaying a square pattern on an area of 32×32 pixels containing a linear phase gradient (see Fig. S2a), while on the remaining area of the SLM, a flat phase is displayed. This phase gradient generates a diffraction spot away from the optical axis, and the light incident onto the remaining area of the SLM collects on the optical axis. We vary the position of the square pattern, d_x and d_y, across the entire area of the SLM and measure the intensity of each diffraction spot, $|A_{\mathrm{SLM}}(d_x, d_y)|^2$, on the camera, and as a result, the intensity profile of the laser beam across the SLM is reconstructed (Fig. S2b)[S2]. The position of the square is varied on an equally spaced grid using 64×64 measurements. The diffraction angle of the linear phase gradient is $\alpha_x = \alpha_y = 0.5^\circ$ both in x- and y-direction. Initially, the square is displayed at the centre of the SLM and a Gaussian is fitted to the resulting diffraction spot on the camera, in a square region of interest of 300 camera pixels. The intensity of each spot is calculated as the sum of all pixel values in the region of interest.

To measure the constant phase, the position of a square sample phase pattern is varied across the entire area of the SLM, similar to our scheme used to measure the intensity. In addition, a reference square pattern is displayed at the centre of the SLM (see Fig. S3a). The beams originating from the two phase patterns interfere at the camera, causing sine-shaped fringes. The spatial phase, ϕ_M, of this interference pattern is detected by fitting a 2D sine pattern to the camera image[S1]

$$I_{\mathrm{IMG}}(x,y) = A^2 + B^2 + 2AB\cos\left[k\left(x\sin\gamma_x + y\sin\gamma_y\right) + \phi_M\right], \tag{S1}$$

where $\gamma_x = \arctan(d_x/f)$ and $\gamma_y = \arctan(d_y/f)$. Here, d_x and d_y are the position of the sample pattern with respect to the reference pattern and f is the focal length of the Fourier lens. A and B are the amplitudes of the diffracted beams caused by the reference and the sample square pattern, respectively. Assuming perfect positioning of the lens at $z = f$ and the camera at $z = 2f$ and assuming a thin and parabolic lens, the measured phase, ϕ_M, corresponds to the phase difference between the reference aperture and the sampling aperture $\varphi_C = \phi_M$. The parameters A, B and ϕ_M are fitted while γ_x and γ_y are calculated.

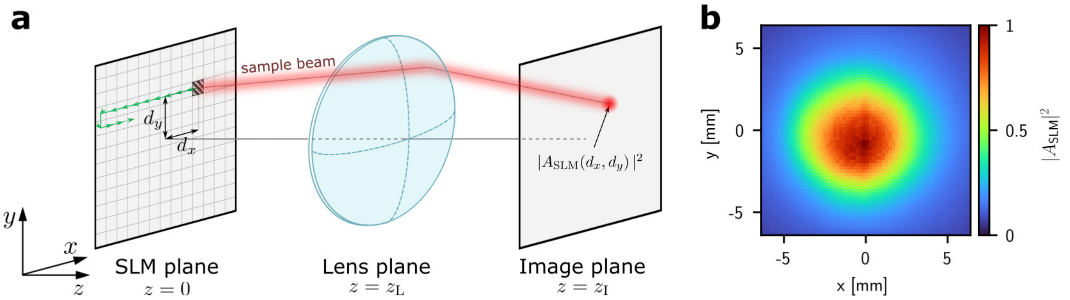

Figure S2. (a) Scheme illustrating the measurement of the laser intensity profile by displaying a series of apertures containing a linear gradient on the SLM[S2] (Fig. adapted from Zupancic et al.[S1]). (b) Resulting laser intensity profile.

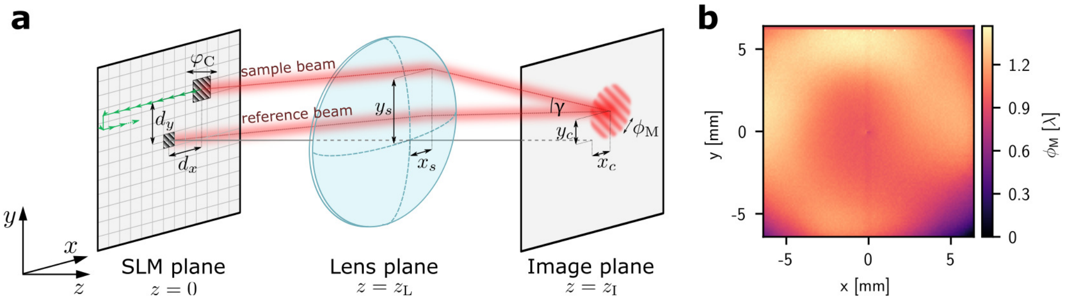

Figure S3. (a) Scheme illustrating the measurement of the constant phase at the SLM using an interferometric approach by displaying a sequence of patterns on sub-regions of the SLM (adapted from Zupancic et al.[S1]). (b) Resulting measured phase, $\varphi_{\rm M}$, expressed in units of λ.

Due to the Gaussian shape of the beam incident onto the SLM, the intensity of the light at the SLM drops off significantly towards the edges. This causes the intensity of the sampling beam B to become very small compared to A as the sampling aperture moves away from the centre of the SLM, resulting in a low contrast $2AB$ of the interference pattern and a poor fit. To counteract this, the size of the sampling patch is increased as it moves away from the centre of the SLM to keep the power contained in the sampling aperture equal to the power contained in the reference aperture. This increases the contrast of the interference pattern on the camera and improves the measurement of the phase at darker regions of the SLM. We use 124×124 measurements, equally spaced across the SLM with a reference phase pattern of 16×16 SLM pixels, resulting in the measured constant phase $\varphi_{\rm C}$ in Fig. S3b. Displaying $-\varphi_{\rm C}$ on the SLM and re-running the measurement results in a flat phase of $\sim \lambda/40$ RMS error. As the measured intensity and phase have 32×32 and 124×124 data points, they are up-scaled to the native resolution of the SLM (central 1024×1024 pixels) using fourth-order Lanczos interpolation[S3]. Before up-scaling, the phase is unwrapped and both measurements are smoothed using a 3×3 uniform filter. It takes approximately 30 minutes to calibrate the intensity pattern and 2 hours to obtain the phase calibration.

Angular spectrum method

We implement the ASM to simulate the propagation of light in our CG minimisation. First, the electric field at the SLM plane, $E_{\rm SLM}(x,y)$, is propagated to the lens plane and multiplied by the aperture, $A_{\rm L}(x,y)$, and phase, $\phi_{\rm L}(x,y)$, of the lens using the relation[S4]

$$E(x,y,z_{\rm L}) = \mathscr{F}^{-1}\left\{\mathscr{F}\left\{E_{\rm SLM}(x,y)\right\} H\left(\kappa'_x, \kappa'_y, z_{\rm L}\right)\right\} A_{\rm L}(x,y) \exp\left[i\phi_{\rm L}(x,y)\right]. \tag{S2}$$

Here, κ'_x and κ'_y are the spatial frequencies, $E\left(x,y,z_{\rm L}\right)$ is the electric field in the lens plane just after the lens and $z_{\rm L}$ is the distance between the SLM plane and the lens plane. $A_{\rm L}(x,y) = circ(r)$ is the circular aperture of the lens with radius r and $\phi_{\rm L}(x,y)$ is the phase delay caused by the lens. The transfer function, $H\left(\kappa'_x, \kappa'_y, \Delta z\right)$, is given by[S4]

$$H\left(\kappa'_x, \kappa'_y, \Delta z\right) = \begin{cases} \exp\left[2\pi i \frac{\Delta z}{\lambda}\sqrt{1-(\lambda\kappa'_x)^2-\left(\lambda\kappa'_y\right)^2}\right] & \text{if } \sqrt{\kappa'^2_x+\kappa'^2_y} < \frac{1}{\lambda}. \\ 0 & \text{otherwise.} \end{cases} \tag{S3}$$

with propagation distance, Δz. The resulting electric field, $E(x,y,z_{\mathrm{L}})$, is then propagated to the image plane using[S4]

$$E(x,y,z_{\mathrm{I}}) = \mathscr{F}^{-1}\left\{\mathscr{F}\left\{E(x,y,z_{\mathrm{L}})\right\} H\left(\kappa_x', \kappa_y', z_{\mathrm{I}} - z_{\mathrm{L}}\right)\right\}, \tag{S4}$$

where $E(x,y,z_{\mathrm{I}})$ is the resulting electric field in the image plane and $\Delta z = z_{\mathrm{I}} - z_{\mathrm{L}}$ is the distance between the lens plane and the image plane (see Fig. 1a).

Using the ASM instead of the Fourier transform enables us to model the lens accurately. Specifically, we use a doublet lens with three spherical surfaces (Thorlabs ACT508-250-B) which causes a phase delay[S4]

$$\phi_{\mathrm{L}}(x,y) = \frac{2\pi}{\lambda}\left[\Delta_{12}(x,y)(n_1-1) + \Delta_{23}(x,y)(n_2-1)\right], \tag{S5}$$

where Δ_{12} and Δ_{23} are the lens thicknesses and $n_1 = 1.59847$ and $n_2 = 1.76182$ the refractive indices of the crown and the flint glass[S5, S6], respectively. The lens thicknesses are given by[S4]

$$\Delta_{ab}(x,y) = -R_a\left(1 - \sqrt{1 - \frac{x^2+y^2}{R_a^2}}\right) + R_b\left(1 - \sqrt{1 - \frac{x^2+y^2}{R_b^2}}\right) \tag{S6}$$

with the radii of the spherical surfaces $R_1 = 137.7\,\mathrm{mm}$, $R_2 = -R_1$ and $R_3 = -930.4\,\mathrm{mm}$. The phase of the doublet, $\phi_{\mathrm{L}}(x,y)$, deviates from the idealised phase of the lens[S4]

$$\phi_{\mathrm{Q}}(x,y) = -\frac{\pi}{\lambda f}\left(x^2+y^2\right), \tag{S7}$$

with the focal length, $f = 250\,\mathrm{mm}$, by $2.8\,\lambda$ (peak-to-valley) across the aperture of the lens (48.3 mm).

In our numerical implementation, we pad the array representing the SLM field with zeros to match the size of the SLM plane with the aperture of the lens used in our experiment. This increases the computational complexity as the matrix size increases from 2048×2048 to 3864×3864. When using the FFT, the matrix representing the SLM plane of 1024×1024 pixels is zero-padded to 2048×2048 pixels, resulting in a pixel spacing $p_{\mathrm{IMG}} = \frac{\lambda f}{2Np_{\mathrm{SLM}}} = 8.32\,\mu\mathrm{m}$ in the image plane, with the number of SLM pixels, N, in each dimension and SLM pixel pitch, p_{SLM}. With the ASM, the pixel size in the SLM plane equals the pixel size in the image plane. To achieve a similar spatial resolution in the output plane using the ASM, each SLM pixel of $12.5\,\mu\mathrm{m}$ size is sub-resolved computationally into 2×2 pixels which increases the number of pixels to 7728×7728. We use a GPU (Nvidia RTX A5000 24 GB) to accelerate our calculations.

ASM wavefront correction

Our method to measure the constant phase, φ_{C}, requires the lens to be parabolic and assumes perfect placement of the lens and the camera. Using equation S1, the measured phase, $\phi_{\mathrm{M}}(x,y)$, includes the phase difference caused by the distorted wavefront at the SLM and the phase differences caused by a non-parabolic lens and a displacement of the camera along the optical axis. As the ASM is capable of modelling the doublet lens and a displaced camera, it is important to separate these phase differences and the wavefront at the SLM, $\phi_{\mathrm{C}}(x,y)$, from each other.

To implement the ASM, we calculate a corrective phase, $\phi_{\mathrm{ASM}}(x,y)$, which only models the phase caused by the displaced, non-parabolic lens and the displaced camera, assuming a flat wavefront at the SLM. To do so, we calculate the path length of every sample beam between the lens and a fixed point in the image plane as well as the phase delay each sample beam collects when passing through the lens, $\phi_{\mathrm{L}}\big(x_s(x), y_s(y)\big)$.

$$\phi_{\mathrm{ASM}}(x,y) = \frac{2\pi}{\lambda}\sqrt{\left[z_{\mathrm{I}} - z_{\mathrm{L}}\right]^2 + \left[x_s(x) - x_c\right]^2 + \left[y_s(y) - y_c\right]^2} + \phi_{\mathrm{L}}\big(x_s(x), y_s(y)\big), \tag{S8}$$

with the position of the sample beam on the lens $x_s(x) = x + z_{\mathrm{L}}\tan(\alpha_x)$ and $y_s(y) = y + z_{\mathrm{L}}\tan(\alpha_y)$, where α_x and α_y are the diffraction angles of the linear phase gradient in x- and y-direction, respectively. The phase is sampled at a point in the image plane with co-ordinates $x_c = f\tan(\alpha_x)$ and $y_c = f\tan(\alpha_y)$. We then subtract the corrective phase pattern, $\phi_{\mathrm{ASM}}(x,y)$, from the measured constant phase to obtain the wavefront at the SLM, $\phi_{\mathrm{C}}(x,y) = \phi_{\mathrm{M}}(x,y) - \phi_{\mathrm{ASM}}(x,y)$.

References

S1. Zupancic, P. *et al.* Ultra-precise holographic beam shaping for microscopic quantum control. *Opt. Express* **24**, 13881–13893, DOI: https://doi.org/10.1364/OE.24.013881 (2016).

S2. Clark, T. W., Offer, R. F., Franke-Arnold, S., Arnold, A. S. & Radwell, N. Comparison of beam generation techniques using a phase only spatial light modulator. *Opt. Express* **24**, 6249–6264, DOI: https://doi.org/10.1364/OE.24.006249 (2016).

S3. Lanczos, C. Trigonometric interpolation of empirical and analytical functions. *J. Math. Phys.* **17**, 123–199, DOI: https://doi.org/10.1002/sapm1938171123 (1938).

S4. Goodman, J. W. *Introduction to Fourier optics* (Macmillan Learning, 2017).

S5. Schott. N-SF11. https://www.schott.com/shop/advanced-optics/en/Optical-Glass/N-SF11/c/glass-N-SF11 (2022).

S6. Schott. N-SK2. https://www.schott.com/shop/advanced-optics/en/Optical-Glass/N-SK2/c/glass-N-SK2 (2022).
